# Cross‐reactivity of hepatitis C virus specific vaccine‐induced T cells at immunodominant epitopes

**DOI:** 10.1002/eji.201444686

**Published:** 2014-10-30

**Authors:** Christabel Kelly, Leo Swadling, Anthony Brown, Stefania Capone, Antonella Folgori, Mariolina Salio, Paul Klenerman, Eleanor Barnes

**Affiliations:** ^1^ Nuffield Department of MedicineUniversity of OxfordOxfordUK; ^2^ OkairosRomeItaly; ^3^ MRC Human Immunology UnitWIMMOxfordUK; ^4^ Oxford NIHR BRC, and Translational Gastroenterology UnitOxfordUK

**Keywords:** Adenovirus, Epitopes, Hepatitis C virus, T cells, Vaccination, Variability

## Abstract

Viral diversity is a challenge to the development of a hepatitis C virus (HCV) vaccine. Following vaccination of humans with adenoviral vectors, we determined the capacity of T cells to target common viral variants at immundominant epitopes ex vivo. We identified two major variants for epitopes NS3_1073_ and NS3_1446_, and multiple variants for epitope NS3_1406_ that occurred in >5% of genotype 1 and 3 sequences at a population level. Cross‐reactivity of vaccine‐induced T cells was determined using variant peptides in IFN‐γ ELISPOT assays. Vaccine‐induced T cells targeted approximately 90% of NS3_1073_ genotype 1 sequences and 50% of NS3_1446_ genotype 1 and 3 sequences. For NS3_1406,_ 62% of subtype‐1b sequences were targeted. Next, we assessed whether an in vitro priming system, using dendritic cells and T cells from healthy donors, could identify a variant of NS3_1406_ that was maximally cross‐reactive. In vitro priming assays showed that of those tested the NS3_1406_ vaccine variant was the most immunogenic. T cells primed with genotype 1 variants from subtype 1a or 1b were broadly cross‐reactive with other variants from the same subtype. We conclude that immunization with candidate HCV adenoviral vaccines generates cross‐reactive T cells at immunodominant epitopes. The degree of cross‐reactivity varies between epitopes and may be HCV‐subtype specific.

## Introduction

Hepatitis C virus (HCV) is a major global pathogen infecting over 180 million people worldwide and is a leading cause of chronic liver disease and malignancy 1. The development of new oral therapies represents a major advance in the field but these are expensive, and not available worldwide 2. An effective prophylactic HCV vaccine would represent a major step forwards in the endeavor to achieve global eradication.

A significant body of evidence shows that HCV viral control is mediated through T‐cell immunity in natural infection, providing the rationale for the development of a T‐cell HCV vaccine 3, 4, 5. However, one of the major challenges facing the development of HCV vaccines is viral genomic diversity within and between hosts 6. To prevent persistent infection, vaccine‐induced T cells may need to target antigens that are conserved between viral strains, or generate cross‐reactive T cells with the capacity to target multiple viral variants.

The assessment of T‐cell cross‐reactivity in natural history studies is complicated by the fact that patients are exposed to multiple unknown viral variants during primary infection and also during viral evolution in chronic disease 7, 8. However, recent studies of the first prophylactic HCV T‐cell vaccine simplified the evaluation of T‐cell cross‐reactivity generated in response to a known, single HCV antigen in healthy human volunteers 9. In these studies, we generated an HCV vaccine based on adenoviral (Ad) vectors, one derived from the chimpanzee (ChAd3) and the second from a rare human serotype (Ad6), encoding the nonstructural HCV genotype 1b protein (NS3‐NS5b), and showed that this technology generates a high magnitude of durable T‐cell responses, which target multiple HCV antigens 9. Using peptides corresponding to the vaccine immunogen, we observed that CD8^+^ T‐cell responses to three epitopes were dominant; NS3_1406_ KLSGLGINAV, NS3_1073_ CVNGVCWTV restricted by human leucocyte antigen (HLA) class‐I A2 alleles, and NS3_1436_ ATDALMTGY restricted by HLA‐A1 alleles. In particular, T‐cell responses to the NS3_1406_ KLSGLGINAV epitope in some cases represented up to 8% of total CD8^+^ T cells following vaccination.

In this study, we evaluate the viral variability of these three epitopes at a population level, and the capacity of Ad vaccine‐induced T cells to cross‐react with high‐frequency epitope viral variants. We show that the immunodominant NS3_1406_ KLSGLGINAV is highly variable at a population level, but that vaccine‐induced T cells may target a subset of common NS3_1406_ viral variants. We also developed an in vitro priming assay to investigate the immunogenicity and cross‐reactive capacity of distinct NS3_1406_ viral variants, for potential inclusion in future HCV vaccine immunogens.

## Results and discussion

### The diversity of dominant HCV epitopes at a population level

**Figure 1:**
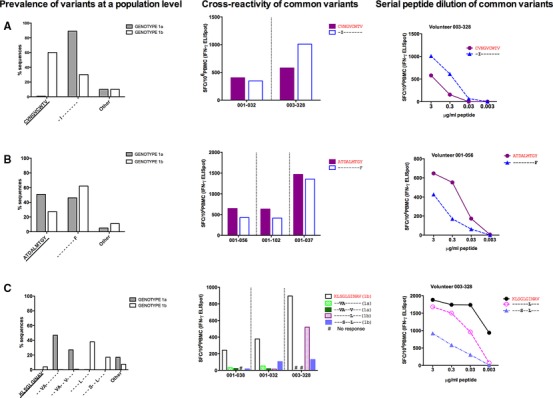
Cross‐reactivity of vaccine‐induced T cells at dominant epitopes. Each row represents a different T‐cell epitope (A) NS3_1073_CVNGVCWTV, (B) NS3_1446_ATDALMTGY, (C) NS3_1406_KLSGLGINAV. Left column: Prevalence of epitope variants at a population level. The amino acid sequence contained within the vaccine immunogen (underlined) and sequence variants present in >5% genotype 1 sequences (dashed lines, amino acid identical to the immunogen) are shown in relation to the frequency of variants at a population level (from Los Alamos Database; number of viral sequences: median genotype 1a/1b: 2313, range 1526–3823; median genotype 3: 488, range 257–640). Genotype 1a: shaded, genotype 1b: unshaded. Middle column: Cross‐reactivity of common variants. The magnitude of T‐cell responses (ex vivo IFN‐γ ELISPOT) to different HCV peptide variants (cross‐reactivity) is shown in healthy volunteers after adenoviral vaccination. Patient numbers are listed under each column (*x*‐axis). The vaccine immunogen sequence is given (red font). Data shown are mean of stimulations performed in duplicate or triplicate for two to three individuals. Right column: Serial peptide dilution of common variants. Peptide serial dilution assays assessed ex vivo in healthy volunteers after adenoviral vaccination, measured by IFN‐γ ELISPOT. Data shown are mean of stimulations performed in duplicate or triplicate for a single volunteer.

First, we evaluated the variability of epitopes NS3_1073_ CVNGVCWTV, NS3_1436_ ATDALMTGY, and NS3_1406_ KLSGLGINAV at a population level, using all reported HCV genotypes 1a, 1b, and 3a sequences derived from the Los Alamos database. This analysis revealed only two prevalent (>5%) sequence variants at each of the epitopes NS3_1073_ CVNGVCWTV and NS3_1436_ ATDALMTGY. These data were confirmed by analysis of our local cohort (data not shown). At epitope NS3_1073_ CVNGVCWTV represented 60% and CINGVCWTV 30% 1b variants, while CINGVCWTV accounted for >90% of 1a variants. Together these two variants were found in >90% of genotype 1 sequences. Similarly at NS3_1436_ ATDALMTGY and ATDALMTGF together accounted for 93% of genotype 1 sequences (Fig. 1A and B, left column). In contrast, epitope NS3_1406_ KLSGLGINAV was highly variable, with no single sequence present in >25% genotype 1 infected individuals (Fig. 1C, left column). We observed that the Ad vaccine immunogen contained an NS3_1406_ variant KLSGLGINAV present in only 4.3% of genotype 1b and undetectable in genotype 1a sequences. The prevalent genotype 1 NS3_1073_ and NS3_1406_ variants are not present in genotype 3 sequences, whereas the NS3_1436_ variants are represented in HCV genotype 3 (Supporting Information Fig. 1A).

These epitopes are previously described in natural infection studies 4, 10, 11 though there is minimal analysis of variability within these epitopes at a population level. The two major variants at epitope NS3_1436_ ATDALMTGY and ATDALMTG**F**, with the latter representing a viral escape variant in HLA‐A1‐positive patients, have been described in prospective studies of primary infection and in cross‐sectional studies of chronic disease 4, 11. T‐cell responses to both NS3_1073_ CVNGVCWTV and CINGVCWTV variants have been previously described in genotype 1 infection although escape variants at this epitope have not been described 12; T‐cell responses to the genotype 3 variant have not been described in natural HCV infection. The immunodominance of epitope NS3_1406_ is well established in natural HCV infection 13, 14 and evidence of viral escape and immune selection of new variants has been described in acute disease 13, 15. Since the NS3_1406_KLSGLGINAV viral variant contained within the Ad vaccine is found in only a minority of people at a population level, the capacity of vaccine‐induced T cells to cross‐react with other common NS3_1406_ viral variants warranted further investigation.

### Cross‐reactivity of vaccine‐induced T cells against circulating viral variants at dominant epitopes

The cross‐reactivity of vaccine‐induced T cells was tested in IFN‐γ ELISPOT assays, using peptides representing epitope variants found in >5% genotype 1 sequences at a population level (Fig. 1, middle and right column). T cells primed by the NS3_1073_ CVNGVCWTV variant contained within the Ad vaccine immunogen, showed good cross‐reactivity with the NS3_1073_ C**I**NGVCWTV variant, and this was confirmed in peptide dilution assays (Fig. 1A, middle and right columns). Others have shown that T cells primed with peptide vaccines containing the NS3_1073_ C**I**NGVCWTV variant are cross‐reactive with the CVNGVCWTV variant 12. It is likely therefore that a vaccine immunogen that hosts either common NS3_1073_ variant would target the majority of genotype 1 infections, since together these variants circulate in 90% of genotype 1 infections at a population level.

Next, we assessed the cross‐reactivity of the NS3_1436_ ATDALMTGY primed T cells to target the NS3_1436_ ATDALMTG**F** variant, the dominant variants in both genotype 1 and genotype 3 infection (Fig. 1B, left panel, and Supporting Information Fig. 1B). Although these appeared to be cross‐reactive at high peptide concentrations in IFN‐γ ELISPOT assay, (Fig. 1B, middle panel), peptide dilution assays showed reduced cross‐reactivity at all peptide concentrations (3.0–0.003 μg/mL) (Fig. 1B, right panel). The lower peptide affinity of T cells primed with NS3_1436_ ATDALMTGY to the ATDALMTG**F** variant supports the published data showing that the latter represents an escape variant in HLA‐A1‐positive infected patients during natural infection 12. Since these two variants dominate in both genotype 1 and 3 infection, it is likely that a vaccine hosting the NS3_1436_ ATDALMTGY epitope would effectively target this variant, but may have reduced efficacy against the ATDALMTG**F** variant found in approximately 50% of genotype 1 and 60% of genotype 3 infected people.

We next assessed the cross‐reactivity of vaccine‐induced NS3_1406_KLSGLGINAV primed T cells. In three volunteers, vaccine‐induced T cells failed to recognize two common population variants NS3_1406_ KL**VA**LGINAV and NS3_1406_ KL**VA**LG**V**NAV (Fig. 1C, middle column), which together represent 39.6% of genotype 1 sequences and 74% genotype 1a sequences at a population level (Fig. 1C, left column). However, there was evidence of cross‐reactivity to two common genotype 1b sequences; NS3_1406_ KLSGLG**L**NAV (volunteer 003–328) and KLS**S**LG**L**NAV (volunteers 001–032 and 003–328) (Fig. 1C, middle column). Peptide dilution assays confirmed that the affinity of KLSGLGINAV primed T cells was lower for the cross‐reactive variants KLSGLG**L**NAV and KLS**S**LG**L**NAV, suggesting that sequence differences in variants at this epitope may be critical for T‐cell recognition of virus infected cells. (Fig. 1C, right column, Supporting Information Fig. 1B). As expected, since T‐cell epitopes have not been described at NS3_1406_ in natural genotype 3 infection, vaccine‐induced T cells failed to recognize KL**R**G**M**G**L**NAV and **R**L**R**G**M**G**L**NAV peptide variants representing 93.4% of genotype 3 sequences (Supporting Information Fig. 1C).

Our data suggest that while vaccine‐induced T cells would not recognize common genotype 1a or genotype 3 viruses at epitope NS3_1406_, they would recognize a majority (58%) of genotype 1b variants. The interindividual variability in responses to genotype 1b variants may reflect a difference in the naïve T‐cell repertoire between individuals. However, the volunteer who made the strongest response to the vaccine also generated the most cross‐reactive responses suggesting a “threshold effect” for the T cells recognition of lower affinity peptide variants. If this is the case, part of the solution to increasing T cell cross‐reactivity will be to increase the magnitude of T cells generated by vaccination.

### In vitro T‐cell priming by NS3_1406_ peptide variants to assess T‐cell cross‐reactivity

Clearly, immunization using a peptide variant with enhanced cross‐reactivity would be beneficial to a T‐cell vaccine. However, it is not practical to test a series of variant containing vaccines in human studies. We therefore attempted to model this in vitro with the aim of identifying variant sequences that were maximally cross‐reactive. For this, we adapted an in vitro priming model to assess the capacity of different NS3_1406_ peptide variants to prime cross‐reactive T‐cell responses 13. We selected the four NS3_1406_ variants with the highest prevalence at a population level (denoted henceforth as 95B, 95C, 95H, and 95I) and compared these to the variant contained within the vaccine immunogen (95A), using DCs derived from 8 HLA‐A2^+^ donors (Supporting Information Table 1). In each case, we used the variant peptides to prime and then tested cross‐reactivity against the other variants.

### The NS3_1406_ variants induce subtype‐restricted cross‐reactivity

**Figure 2:**
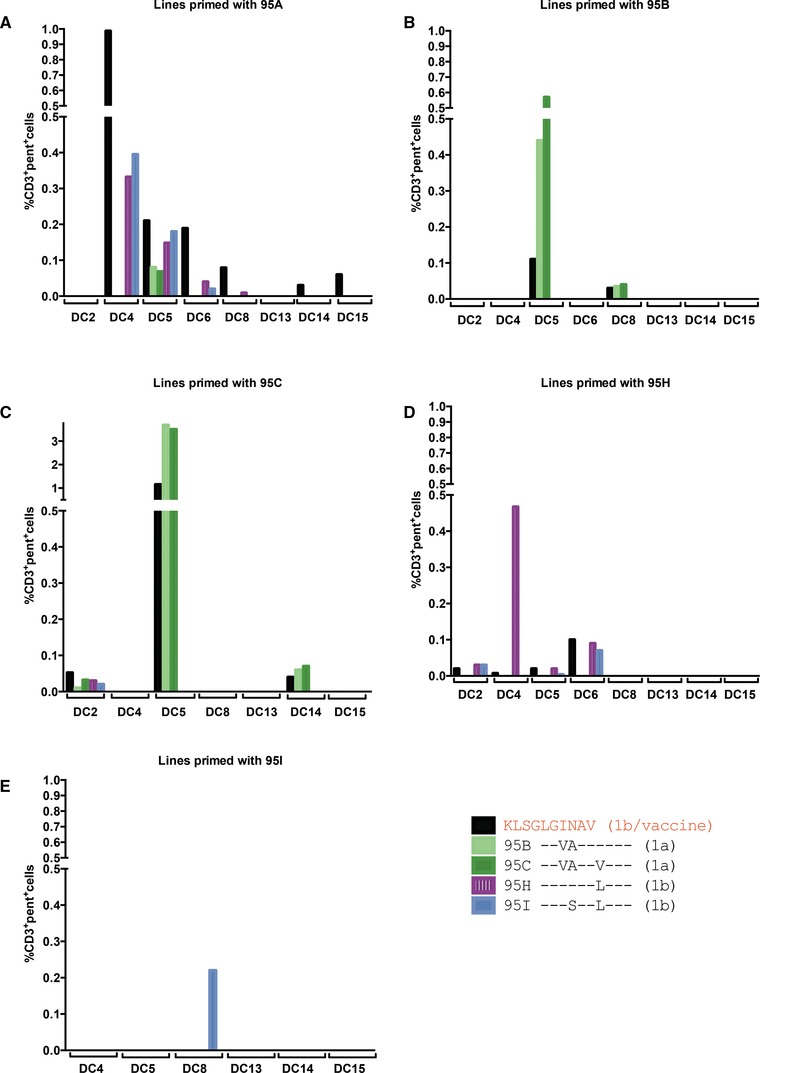
Cross‐reactivity of T‐cell lines primed by NS3_1406_ peptide variants. Naïve T cells were primed from volunteers (labeled “DC”) using NS3_1406_ variants (A) 95A, (B) 95B, (C) 95C, (D) 95H, and (E) 95I. Each T‐cell line generated was stained with multiple NS3_1406_ variant pentamers to assess T‐cell cross‐reactivity. Each graph represents percentage pentamer^+^CD3^+^ cells in T‐cell lines. The vaccine immunogen sequence (95A) is listed in red. Dashes, amino acid identical to 95A. Coloured bars represent the peptide used to prime a given volunteers naïve T cells. Data shown are from six to eight donors, and are representative of six to eight independent experiments, each performed in triplicate with a single donor.

**Figure 3:**
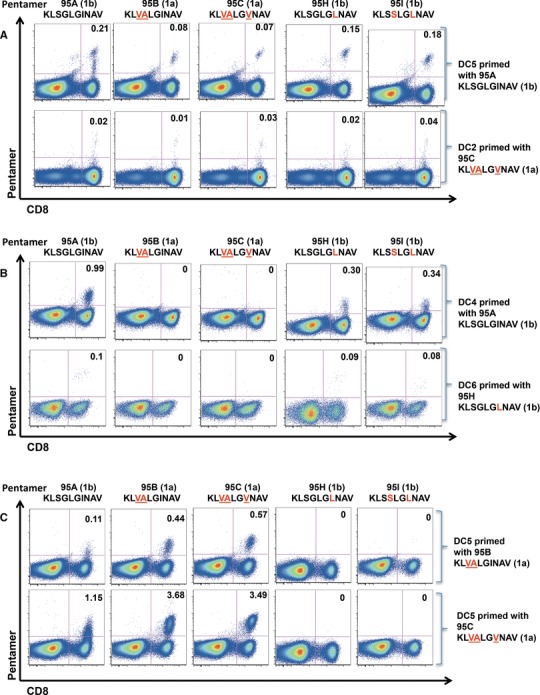
. Cross‐reactivity of T‐cell lines primed by NS3_1406_ peptide variants. Representative flow cytometry plots from four healthy volunteers (DC2, 4, 5, and 6) are shown. Naïve T cells were primed from volunteers using NS3_1406_ variants (95A, B, C, H, and I). Each T‐cell line was stained with multiple NS3_1406_ variant pentamers to assess T‐cell cross‐reactivity. Each plot represents staining with a different pentamer (given at the top of the column). (1a) = Genotype 1a (1b) = Genotype 1b. Percentage pentamer^+^CD3^+^ cells are shown (top right corner of each plot). Cells are gated on live CD3^+^ lymphocytes. Three patterns of cross‐reactivity are shown: (A) Universal cross‐reactivity: Volunteer DC5 primed with 95A (top row) and volunteer DC2 primed with 95C (bottom row) recognize all common sequence variants. (B) Genotype 1b subtype cross‐reactivity: Volunteer DC4 primed with 95A (top row) and volunteer DC6 primed with 95H (bottom row) recognize genotype 1b variants only. (C) Genotype 1a subtype cross‐reactivity: Volunteer DC5 primed with 95B (top row) and 95C (bottom row) recognizes genotype 1a variants.

We succeeded in priming T‐cell responses to all variants tested (Fig. 2). Peptide 95A successfully primed T cells in six of eight individuals (75%, Fig. 2A), 95H in four of eight (50%) volunteers, and 95C in three of seven (42.8%) volunteers tested (representative FACS plots Fig. 3). The magnitude of the responses generated after priming varied between individuals and was greatest for individual DC5 primed with peptide 95C (>3% pentamer^+^/CD3^+^ T cells) (Fig. 3C).

Two variants 95I and 95B primed responses in only one of six and two of eight volunteers, respectively (Fig. 2B and E). It is possible that these variants have reduced T‐cell receptor binding affinities, or that the naïve T‐cell precursor frequencies for these particular variants are low. Since it has previously been shown that the enumeration of naïve T‐cell precursor frequencies is generally conserved between individuals 16, the inclusion of peptides 95I and 95B would represent a poor choice for inclusion in a vaccine immunogen, even if these variants occur commonly in HCV‐infected people.

### HCV NS3_1406_ variants are not all equally cross‐reactive

Next, we assessed the cross‐reactivity of T cells primed in vitro using NS3_1406_ variant‐specific HLA class‐I pentamers and FACS analysis. In general, no single peptide variant had the capacity to generate T cells that were cross‐reactive with all other variants in the majority of individuals. Broad T‐cell cross‐reactivity was observed in two cases only; volunteer DC5 primed with 95A and DC2 primed with 95C (Fig. 2A, C and 3A).

We observed that cross‐reactivity was HCV‐subtype specific; genotype 1b variants generally primed T cells that were cross‐reactive with other 1b variants and genotype 1a variants primed T cells that were cross‐reactive with other 1a variants. For example, three of four lines primed by 95H (1b variant) were cross‐reactive with the other common 1b variants but did not recognize the 1a variants (Fig. 2D and 3B). Similarly, four of five individuals primed with variants 95B or 95C recognized genotype 1a peptides, but not genotype 1b peptides (Fig. 2B, C and 3C). The poorly immunogenic peptide 95I primed T cells in one individual only that did not cross‐react with any other variants (Fig. 2E). Functional analysis by intracellular cytokine staining (ICS) showed that the cross‐reactive responses identified by pentamer staining produced multiple cytokines including IFN‐γ, TNF‐α, IL‐2, CD107a, and MIP‐1β (Supporting Information Fig. 3).

This is the first study to our knowledge, to use an in vitro model to assess the immunogenicity and cross‐reactivity of dominant T‐cell epitope variants for inclusion in a vaccine immunogen. We found that in vitro no single variant was able to prime naïve T‐cell responses in all individuals tested. This most likely reflects diversity in the host TCR repertoire between individuals. Furthermore, no single NS3_1406_ variant was capable of generating T cells that were cross‐reactive with all other variants assessed although cross‐reactivity within subtype was readily achieved. Overall, this data concurs with the ex vivo analysis of donors primed with variant 95A contained within the vaccine.

The generation of a universally cross‐reactive variant at this dominant epitope may not be possible until we better understand the molecular triggers at the TCR/HLA–peptide interface. Once this is achieved, it may be possible to synthesize cross‐reactive variants through rational epitope design. Since cross‐reactive responses were subtype specific, an alternative approach would be to engineer a mosaic immunogen that hosts both genotype 1a and 1b variants. However, in theory inclusion of two variants for a single epitope in a vaccine immunogen may result in competition with one another and so failure to prime strong responses to either variant. An alternative approach is to vaccinate using prime/boost regimens containing different variants of the same epitope and so select a population of T cells with broader T‐cell cross‐reactivity.

Finally, we recognize the limitations of a comparative in vitro culture system that is not reliant on antigen processing and validation of this approach for immunogen design will ultimately require testing in preclinical animal models, and in humans with a physiological T‐cell repertoire.

## Concluding remarks

Ultimately, T‐cell vaccines against pathogens with diverse genomes such as HIV and HCV should aim to generate T cells that target a broad range of HCV antigens restricted by a range of HLA class‐I and class‐II alleles. This will maximize the chances of memory T‐cell populations generated by vaccination recognizing a wide range of incoming viral strains. But in addition, rational immunogen design should take account of the cross‐reactive capacity of individual T‐cell epitopes. This will be particularly important for dominant T‐cell epitopes that target variable parts of the genome. Failure to take account of the latter may result in a dominant pool of T cells that are highly specific for rare sequence variants only. Variant 95A, the variant currently included in the vaccine candidate assessed here, appears to be the most immunogenic of all those tested for the capacity to prime T‐cell responses.

Overall, we conclude that immunization with a candidate HCV adenoviral vaccine encoding a genotype 1b sequence may generate cross‐reactive T cells at immunodominant epitopes, although in the case of epitope NS_1406_ T‐cell cross‐reactivity may be HCV‐subtype restricted.

## Materials and methods

### Participants and vaccination

Healthy volunteers, recruited in Oxford (UK) were vaccinated as previously described with ChAd3 and Ad6 vectors encoding the NS3‐NS5B region of a genotype 1b sequence (ChAd3‐NSmut) at 2.5 × 10^10^ vp dose in prime boost schedules 9 (Supporting Information Table 1). All gave written informed consent. The study was conducted according to the principles of the Declaration of Helsinki and Good Clinical Practice. Samples were randomly selected to reflect a range of magnitude of T‐cell responses to the vaccine (Supporting Information Table 1).

### Epitope Variability

Viral sequences of each epitope were downloaded from Los Alamos database (http://hcv.lanl.gov/content/index) (median genotype 1a/1b: 2313, range 1526–3823; median genotype 3: 488, range 257–640). Single variants with a prevalence of >5% were identified at each epitope.

### IFN‐γ ELISPOT assay

IFN‐γ ELISPOT assays were performed according to manufacturer instructions (Mabtech) on freshly isolated PBMC or thawed PBMC rested overnight. Cells were plated at 2 × 10^5^ PBMC/well in duplicate or triplicate and cocultured with HCV‐specific peptides overnight at 3 μg/mL unless otherwise specified. Internal controls were DMSO, Concavalin A, and FEC (mixed HLA class I‐restricted peptides from influenza, Epstein–Barr virus, and CMV).

### Priming of HCV‐specific T cells in healthy volunteers

Overview described in Supporting Information Fig. 2.

### Generation of immature dendritic cells (adapted from 13, 17)

Whole blood cones from healthy donors from The John Radcliffe Hospital, Oxford, UK were stained with HLA‐A2‐FITC antibody (1:50). PBMC from HLA‐A2^+^ samples were isolated via density gradient separation. Monocytes were purified by positive enrichment using anti‐CD14^+^ magnetic beads (Miltenyi Biotec). The negative fraction was frozen for later use. Immature DCs were generated by culturing monocytes for 4 days at 500 000 cells/mL in 6‐well plates (Costar) in DC medium (Cellgenix) supplemented with Rh10 (10%), IL4 (1000 IU/mL), and GM‐CSF (800 IU/mL) (Peprotech, UK). On day 4, cells were matured with LPS 10 ng/mL (Sigma) and peptide (10 μg/mL) (Proimmune, UK).

On day 6, mature, peptide pulsed DCs were harvested, washed, and replated in 24‐well plates (Costar) at 250 000 cells/mL/well. Autologous thawed PBCMs were added at 1:20 (DC:PBMC) (1 mL of 5 × 10^6^ cells/mL/well). Cells were incubated in complete T‐cell medium (Rh10 + 12.5 mM HEPES (Invitrogen) + 50 μM 2‐mercaptoethanol (Sigma‐Aldrich). IL2 (100 IU/mL, Roche) and IL7 (5 ng/mL, R and D) were added (day 10). Fresh media and cytokines were added every 2–3 days until harvesting.

### Restimulation of cell lines

Seven to nine days after priming, peptide was added to cultured cell lines (10 μg/mL). For the subsequent restimulations, autologous PBMC were thawed and pulsed with peptide for 3 h at 37°C then added to plates at 10 × 10^6^/mL/well.

### Tetramer staining

PBMC were stained with HLA*0201 matched pentamers labeled with phycoerythrin (PE: proimmune) then costained with live/dead antibody (fixible near‐IR dead cell stain kit: life technologies), CD3‐PO (Pacific Orange) and CD8‐PB (Pacific Blue). Cells were analyzed by flow cytometry with a BD LSRII and FlowJo v.9.6.1.

### Intracellular cytokine staining

T‐cell lines were stimulated overnight with single peptides from Supporting Information Table 2 (10 μg/mL) or phorbol 12‐myristate 13‐acetate (PMA)/ionomycin (500 ng/mL) or left unstimulated (controlled for DMSO 50 ng/mL). CD107a‐PeCy5 was added at the start of the stimulation and Brefeldin‐A was added 1 h later. The next day cells were fixed, permeabilized, and stained with: CD3‐PO, IFN‐γ‐Alexa700, MIP1β‐PE, TNFa ‐PECy7, IL2‐ allophycocyanin, CD8 ‐PB, live dead allophycocyanin‐Cy7. Samples were analyzed on a BD LSRII with FlowJo v.9.6.1.

## Statistical analysis

Formal statistical analysis was not performed given the small sample sizes.

## Conflict of interest

A. Folgori is a named inventor on patent applications covering HCV‐vectored vaccines and chimpanzee adenovirus vectors. A.F. and S.C. are employees of Okairos.

**Abbreviations**
Ad: adenoviral

## Supporting information

As a service to our authors and readers, this journal provides supporting information supplied by the authors. Such materials are peer reviewed and may be re‐organized for online delivery, but are not copy‐edited or typeset. Technical support issues arising from supporting information (other than missing files) should be addressed to the authors.

Peer review correspondence

**Figure S1**. Cross–reactivity of genotype 3 HCV epitope variants
**Figure S2**. Schema for in vitro priming of naïve T cells using different HCV sequence variants
**Figure S3**. Representative intracellular cytokine staining of successful T cell lines primed with variants of epitope NS31406
**Figure S4**. Gating strategy for FACs plots
**Table S1**. Vaccination regimen of volunteers whose samples were used in Fig. 1 and Supplementary Information Fig. 1, as described in Barnes et al. 2012 [11].
**Table S2**. Variants of NS31406 used for T cell priming and their relative frequency in genotype 1 HCV infection.
