# Supplementary material for: Cross‐reactivity of hepatitis C virus specific vaccine‐induced T cells at immunodominant epitopes
Source: Eur J Immunol. 2014 Oct 30;45(1):309–16. doi: 10.1002/eji.201444686 (PMC4784727; doi:10.1002/eji.201444686)
Supplement: Supplementary file 2 — Figure S1. Cross–reactivity of genotype 3 HCV epitope variants Figure S2. Schema for in vitro priming of naïve T cells using different HCV sequence variants Figure S3. Representative intracellular cytokine staining of successful T cell lines primed with variants of epitope NS31406 Figure S4. Gating strategy for FACs plots Table S1. Vaccination regimen of volunteers whose samples were used in Fig. 1 and Supplementary Information Fig. 1, as described in Barnes et al. 2012 [11]. Table S2. Variants of NS31406 used for T cell priming and their relative frequency in genotype 1 HCV infection. [file EJI-45-309-s002.pdf]

European Journal of Immunology

Supporting Information
for
DOI 10.1002/eji.201444686

Christabel Kelly, Leo Swadling, Anthony Brown, Stefania Capone, Antonella Folgori, Mariolina Salio, Paul Klenerman and Eleanor Barnes

Cross-reactivity of hepatitis C virus specific vaccine-induced T cells at immunodominant epitopes

© 2014 The Authors. *European Journal of Immunology* published by WILEY-VCH Verlag GmbH & Co. KGaA, Weinheim

Supplementary Fig. 1 Cross-reactivity of genotype 3 HCV epitope variants

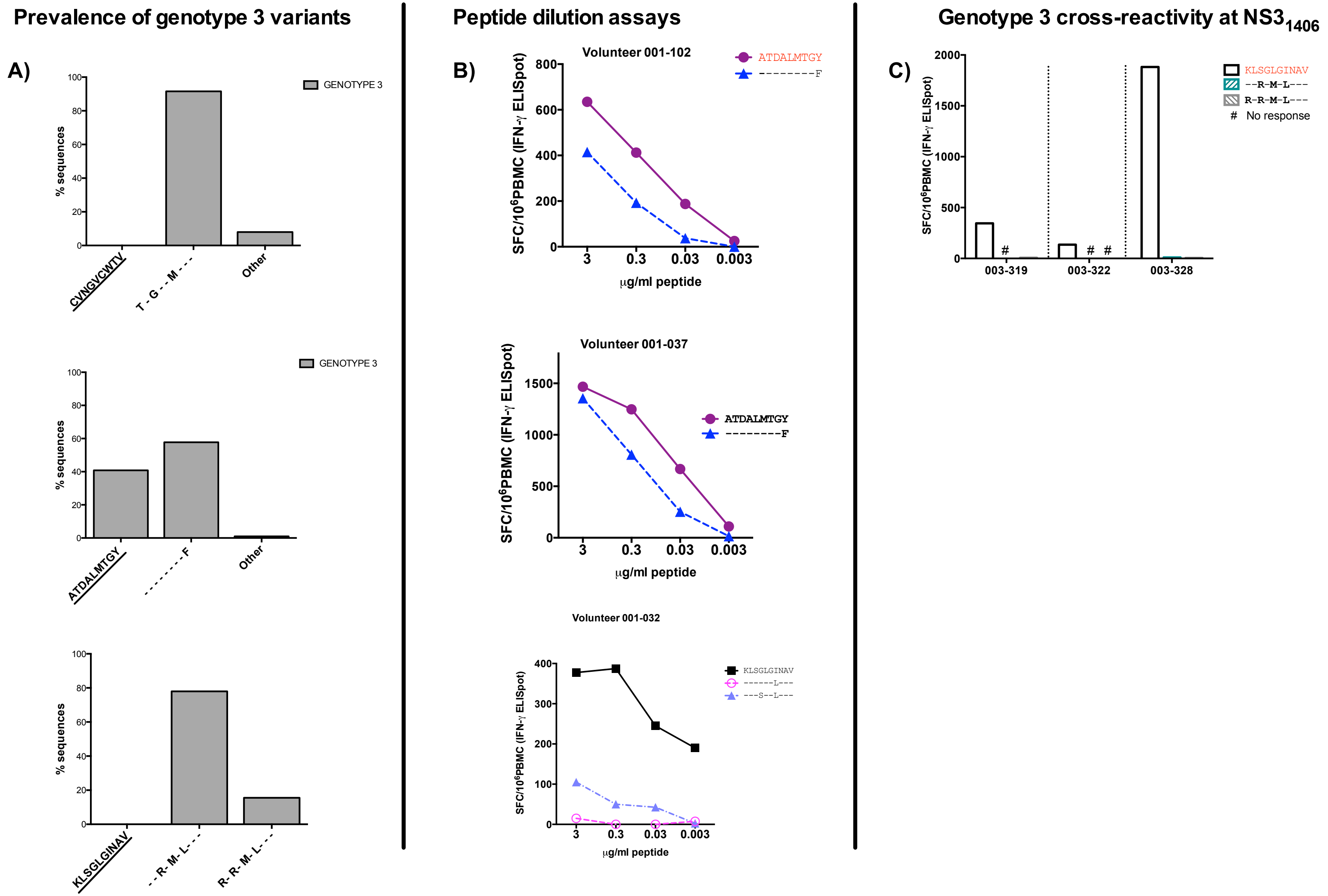

A) Left column: Prevalence of genotype 3 epitope variants at a population level at $NS3_{1073}$CVNGVCWTV, $NS3_{1446}$ATDALMTGY and $NS3_{1406}$KLSGLGINAV. *x* axis: The amino acid sequence contained within the vaccine immunogen (underlined) and sequence variants present in >5% genotype 1 sequences. Dashed line indicates amino acid homology with the immunogen. % frequency at a population level (from Los Alamos Database) is indicated on the *y* axis. Shaded column= genotype 1a , unshaded column=genotype 1b. **B) Middle Column:** Effect of serial dilution on T cell cross-reactivity**.** Each plot represents a different volunteer, vaccinated with the NSmut immunogen, containing the amino acid sequence listed in full at each epitope (see Supplementary Table 1 for vaccination regimen). Magnitude of T cell response is measured by IFNγ-ELISpot, indicated on the *y* axis. Peptide concentration is listed on the *x* axis. **C) Right Column:** Cross-reactivity of vaccine induced T cells in healthy volunteers to common genotype 3 HCV $NS3_{1406}$ variants. The vaccine immunogen sequence is in red type. Each peptide is represented by a different column. Magnitude of T cell response is measured on the *y axis* by IFNγ-ELISpot. Volunteers 003-319, 003-322 and 003-328 *(x axis)* received ChAd3 prime.

Supplementary Fig.2

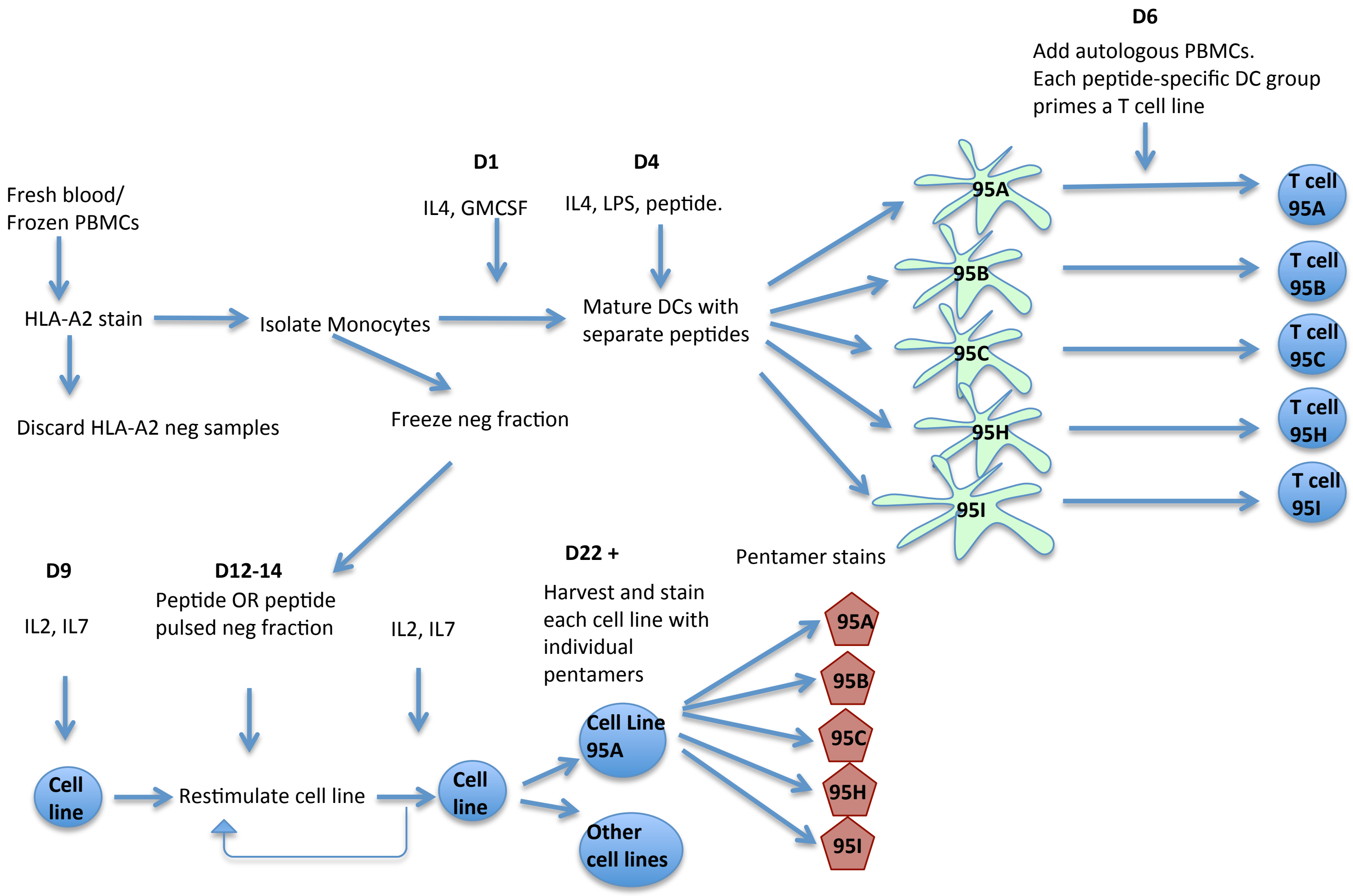

Supplementary Fig. 2 Schema for in vitro priming of naïve T cells using different HCV sequence variants

HLA-A2 positive samples were selected for monocyte isolation. Monocyte depleted fraction ("neg fraction") was frozen and reserved for later use. DCs were matured with one of 5 individual peptides, matched to sequence variants in the HCV genome named 95A, 95B, 95C, 95H and 95I. DCs then prime naïve T cell lines which were restimulated with peptides or peptide pulsed autologous PBMC. Each T cell line was stained with 5 pentamers matched to the individual sequence variants (95A-I). neg=negative D=Day DC=dendritic cell IL= interleukin LPS= lipopolysaccharide

Supplementary Fig.3

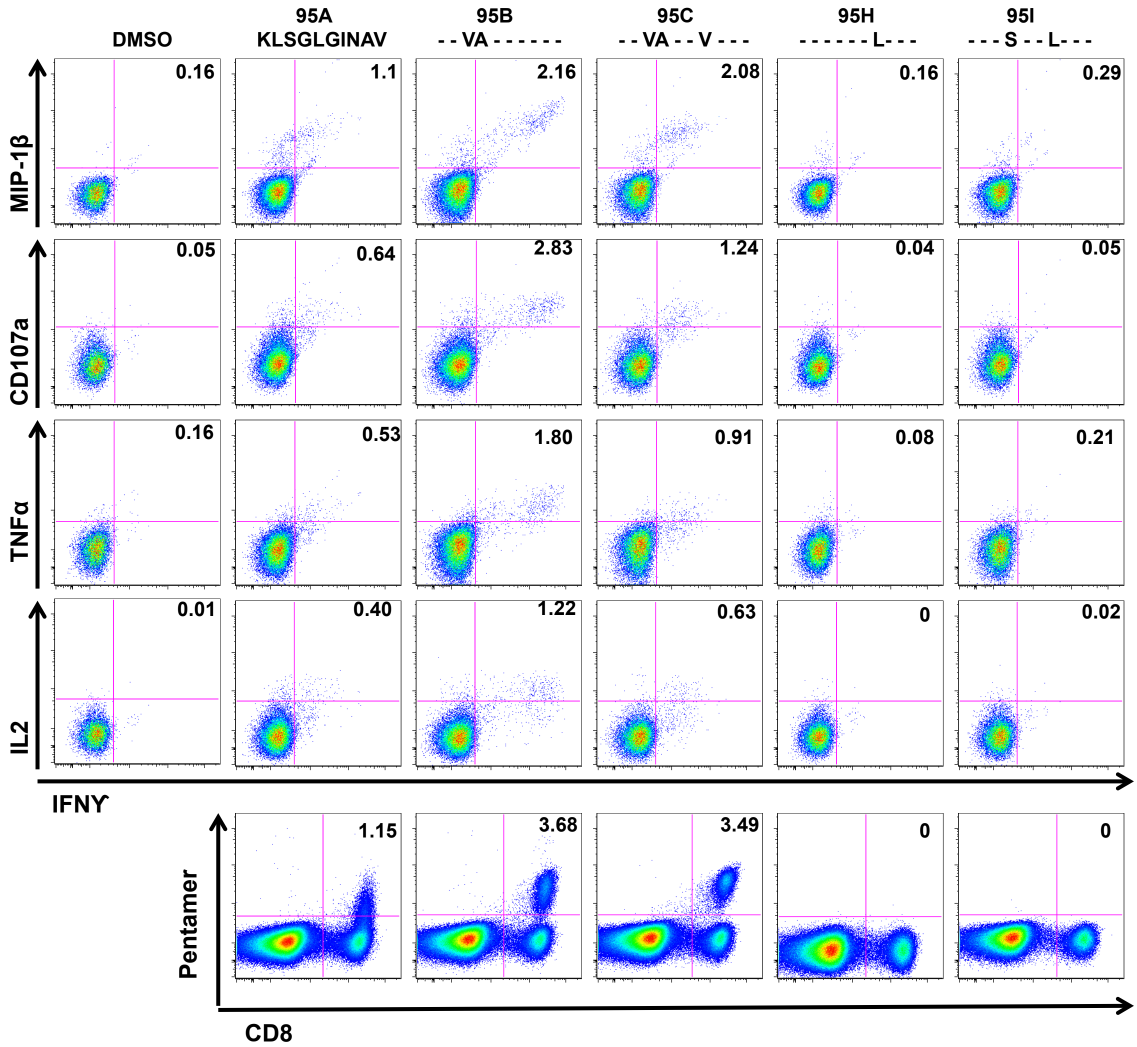

Representative intracellular cytokine staining of successful T cell lines primed with variants of epitope $NS3_{1406}$

The top 4 rows represent ICS staining. The bottom row represents comparative pentamer staining. A cell line from volunteer DC5 was primed by peptide 95C and stimulated with different variants of epitope $NS3_{1406}$ (95A, 95B, 95C,95H and 95I listed above each column) then stained for cytokines (*y axis*). Amino acid homology to the peptide contained within the vaccine immunogen is indicated by a dashed line. Cells are gated on live $CD8^+$ cells. % $CD8^+$cells positive for both IFNγ and the *y axis* cytokine is indicated in the top right of each plot.

Supplementary Fig.4

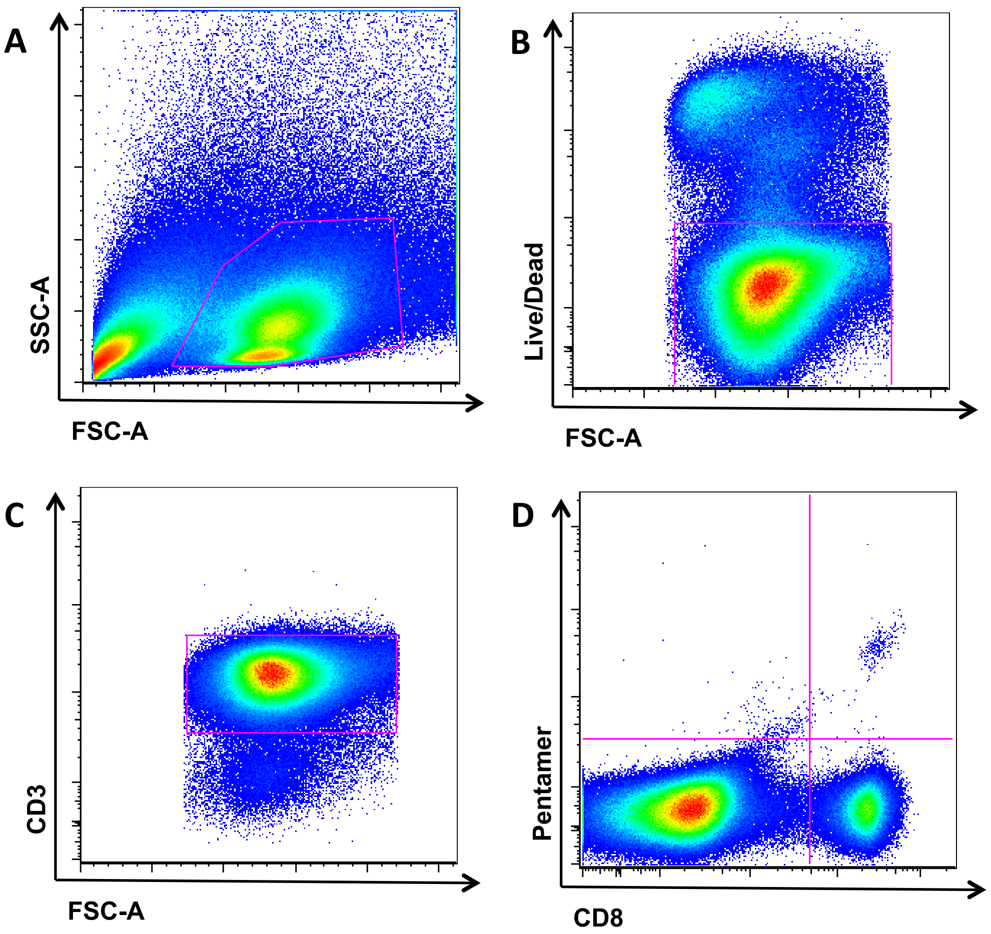

Gating strategy for FACs plots

Sequential gating is shown (A-D). Cells were gated on **A**) lymphocytes, **B**) live cells, **C**) CD3+ cells **D**) CD8+ pentamer+ cells.

Supplementary Table 1

Vaccination regimen of volunteers whose samples were used in Fig. 1 and Supplementary Information Fig. 1, as described in Barnes et al. 2012 [11]. All volunteers received vaccination at 2.5 X 10^{10}vp/ml. Samples were taken post priming vaccination. One individual (001-032) was also sampled post boost.

Total T cell response using peptides spanning the vaccine immunogen is given measured by IFNγ ELISpot assay. The response to the NS3 protein alone is given in parenthesis.. Vp= viral particles. Wk=week of vaccination, ChAd3= chimpanzee adenovirus 3, Ad6= human adenovirus 6. SFC=spot forming colonies PBMC=peripheral blood mononuclear cell

**Volunteer**	**Figure**	**Prime (Week)**	**Boost**	**Trial Week in assay**	**Total IFNγ SFC/10^6 PBMC (NS3 IFNγSFC /10^6 PBMC)**
**001-038**	Fig.1C	ChAd3-NSmut Wk 0, Wk4	n/a	6	735 (706)
**001-032**	Fig.1C	ChAd3-NSmut Wk 0, Wk4	Ad6-NSmut (Wk14)	26	1561 (1447)
**001-032**	Fig.1A	ChAd3-NSmut Wk 0, Wk4	n/a	8	2325 (2064)
**001-056**	Fig.1B	ChAd3-NSmut (Wk0)	n/a	2	1223 (509)
**001-102**	Fig.1B	ChAd3-NSmut Wk 0, Wk4	n/a	6	1133 (902)
**001-037**	Fig.1B	ChAd3-NSmut Wk 0, Wk4	n/a	6	2435 (1480)
**003-328**	Fig.1A, Fig.1C, Supp Fig.1	ChAd3-NSmut (Wk0)	n/a	8	2220 (1682)
**003-319**	Supp Fig.1	ChAd3-NSmut (Wk0)	n/a	4	368 (330)
**003-322**	Supp Fig.1	ChAd3-NSmut (Wk0)	n/a	2	1442 (1007)

Supplementary Table 2

Variants of $NS3_{1406}$ used for T cell priming and their relative frequency in genotype 1 HCV infection.

Based on analysis of 1526 HCV sequences from Los Alamos database (hcv.lanl.gov/). "– " indicates amino acid identity with the prototype sequence in the vaccine immunogen listed in full.

Sequence	% 1a	% 1b	% Genotype 1 HCV overall	Peptide name
**KLSGLGINAV**	0	4.3	2.0	95A
**--VA------**	46.8	0	24.8	95B
**--VA--V---**	27.0	0.6	14.5	95C
**------L---**	0.2	38.4	18.2	95H
**---S--L---**	0	19.6	9.2	95I
