# Supplementary material for: Cross‐reactivity of hepatitis C virus specific vaccine‐induced T cells at immunodominant epitopes
Source: Eur J Immunol. 2014 Oct 30;45(1):309–16. doi: 10.1002/eji.201444686 (PMC4784727; doi:10.1002/eji.201444686)
Supplement: Supplementary file 1 — Peer review correspondence [file EJI-45-309-s001.pdf]

Peer review correspondence

European Journal of Immunology

Cross-reactivity of hepatitis C virus-specific vaccine induced T cells at immunodominant epitopes

Christabel Kelly, Leo Swadling, Anthony Brown, Stefania Capone, Antonella Folgori, Mariolina Salio, Paul Klenerman and Eleanor Barnes

Correspondence: Dr. Eleanor Barnes, Peter Medawar Building, South Parks Rd, Oxford, OX1 3SY, UK

Review Timeline:	Submission date:	24 March 2014
	First Editorial decision:	13 May 2014
	First Revision received:	2 August 2014
	Accepted:	24 September 2014

Handling Executive Committee member: Prof. Annette Oxenius

Please note that the correspondence below does not include the standard editorial instructions regarding preparation and submission of revised manuscripts, only the scientific revisions requested and addressed.

<u>First Editorial Decision – 13 May 2014</u>

Dear Dr. Barnes,

Please accept my sincere apologies for the prolonged delay in processing the review of your Manuscript ID eji.201444686 entitled "CROSS-REACTIVITY OF HCV SPECIFIC VACCINE INDUCED T-CELLS AT IMMUNODOMINANT EPITOPES", which you submitted to the European Journal of Immunology has been reviewed. There was a severely delayed report and a difference in opinion for which we sought additional advice. All opinions have now been received and the comments of the referees are included at the bottom of this letter.

A revised version of your manuscript that takes into account the comments of the referees will be reconsidered for publication.

You should also pay close attention to the editorial comments included below. In particular, please edit your figure legends to follow Journal standards as outlined in the editorial comments. Failure to do this will result in delays in the re-review process.

Please note that submitting a revision of your manuscript does not guarantee eventual acceptance, and that your revision will be re-reviewed by the referee(s before a decision is rendered.

If the revision of the paper is expected to take more than three months, please inform the editorial office. Revisions taking longer than six months may be assessed by new referee(s) to ensure the relevance and timeliness of the data.

Once again, thank you for submitting your manuscript to European Journal of Immunology and we look forward to receiving your revision.

Yours sincerely,
Laura Soto Vazquez

On behalf of
Prof. Annette Oxenius

Editorial Office
European Journal of Immunology
e-mail: ejied@wiley.com
www.eji-journal.eu

****

Reviewer: 1

Comments to the Author
The authors investigated the capacity of T cells to target common viral variants at 3 immundominant HCV epitopes ex-vivo and used an in-vitro DC priming model to assess the capacity of NS31406 variants to generate cross-reactive T-cells.
They showed cross-reactivity of vaccine induced T-cells for the 3 epitopes. Vaccine induced T-cells targeted the mutant epitope peptides found in approximately 90% of NS31073 genotype-1 sequences, 50% of NS31446 genotype-1 and 3 sequences and up to 62% of NS31406 genotype-1b sequences. In-vitro priming assays showed that the NS31406 vaccine variant was the most immunogenic. T-cells primed with genotype-1 variants from subtype 1a or 1b were broadly cross-reactive with other variants from the same subtype.
The study used vaccine-induced T cells are very interesting and valuable for HCV vaccine development, but the data of DC priming model are limited for evaluation of epitopes since the T cells were induced in the samples from one or two individuals.

Major comments:

1. Fig1C: only one subject showed the cross-reactivity with one variant (not two). This is quite different from other epitopes. The titration data showed the different spots between these variants (may be significant, no statistics analysis), suggesting that this difference may be critical in T cell recognition of virus-infected cells.

2. The evidence of cross-reactive T cell induction in the DC priming experiment is weak because they were induced in only 2-3 for 95A and 1-2 for others.

Minor comments:

1. The method in Fig2 is not clear. ICS assay?

Reviewer: 2

Comments to the Author

This paper discusses an extremely important issue in viral vaccine field, the cross-reactivity. The authors investigated the cross reactive potential of T cells induced by an HCV vaccine based on adenoviral (Ad) vectors, focusing on three previously described epitopes. The spread of this kind of approach is necessary to improve the coverage of vaccines against viruses that present a high diversity of protein sequences and, consequently, of epitopes. The experiments were adequately conducted and the obtained results demonstrate that the vaccine seems to provide good coverage for common populations variants from the epitope region NS31073 CVNGVCWTV in the immunized individuals. The NS31436 ATDALMTGY and NS31406 KLSGLGINAV epitopes do not stimulated a desirable cross-reactive response, mainly in relation to the NS31406 epitope, even considering the demonstrated immunogenicity in the variant contained in the vector (95A). The authors raised questions about future strategies as the use of both variants for NS1436 Y/F or the synthesis of cross-reactive NS31406 variants through rational epitope design to improve coverage and the efficiency of immunogens and its possible consequences in loss of immunogenicity.

I just have two comments to share with you:

On page 5, line 2, the epitope ATDALMTGY is duplicated.

I recommend the use of identity rather than homology to describe that two positions in a sequence are shared.

European Journal of Immunology

Reviewer: 3

Comments to the Author

This is a follow-up study of the authors' 2012 Science Translation Medicine report on a phase 1 trial of the adenovirus-based HCV vaccines to induce broad and sustained T cell responses. HCV is genetically variable and the induction of functional cross-reactive T cells will be essential in any working vaccines. In this study, through database search and several in vitro assays, the authors investigated how viral genotypic and epitope sequence differences might influence the effectiveness of the vaccine. The authors first used several clinical samples from the STM study to show cross-reactive T cells to three NS3 epitopes were induced by vaccination. They then developed a scheme to stimulate naïve T cells in normal blood donors in vitro with the NS3 1406 peptide and variants, and observed cross-reactive T cells were elicited in some cases (~30%). Based on the results, the authors conclude that immunization with the candidate AdV-based HCV vaccines can generate cross-reactive T cells at immunodominant epitopes. This reviewer is not certain if the results directly support the conclusion. Vaccination with the AdV-based vaccine is a complex process involving successful delivery, expression, processing and presentation of the viral antigens, while the in vitro assays subjected the T cells to non-physiological conditions (possible overdose of cytokines, peptides and DC to T cell ratio). Even so, the conditions used in the in vitro assays showed only a fraction (<50%) of the blood samples produced cross-reactive T cells. The reviewer found the in vitro assays intriguing but the link of the results to the AdV-HCV vaccine unconvincing. In another word, it may be more useful if the authors focus on the in vitro assays and the particular NS3 epitopes, and explain why they works only in some samples; Or the authors refocus to define and analyze cross-reactive T cells and T cell epitopes in the vaccines from the 2012 STM study.

A number of areas in this manuscript can be improved:

(1) What are the criteria for selection/inclusion of the particular clinical samples in this study? Have they been prescreened for high T cell responses?

(2) Supplementary Table 1 should be converted to a standard table, and ELISpot data (total IFN-SFC and NS3-specific IFN-SFC be included in the table. This is important for understanding the other data.

(3) The Los Alamos database used by the authors to analyze HCV epitope diversity is outdated. Recent virus sequences should be included in the analysis to ensure the epitopes used represent circulating viruses.

(4) It seems unsatisfactory that the results and discussion are limited to the peptide sequences involved (or no involved) in the induction of cross-reactive T cells. So much is known about antigen presentation on MHC and TCR and it will be useful to interpret and discuss the data whether the peptides and cross-reactive variants follow the general rules in antigen presentation, or not and the explanation.

European Journal of Immunology

European Journal of Immunology

First Revision – authors' response – 2 August 2014

Reviewer: 1

Comments to the Author

The authors investigated the capacity of T cells to target common viral variants at 3 immunodominant HCV epitopes ex-vivo and used an in-vitro DC priming model to assess the capacity of NS31406 variants to generate cross-reactive T-cells.

They showed cross-reactivity of vaccine induced T-cells for the 3 epitopes. Vaccine induced T-cells targeted the mutant epitope peptides found in approximately 90% of NS31073 genotype-1 sequences, 50% of NS31446 genotype-1 and 3 sequences and up to 62% of NS31406 genotype-1b sequences. In-vitro priming assays showed that the NS31406 vaccine variant was the most immunogenic. T-cells primed with genotype-1 variants from subtype 1a or 1b were broadly cross-reactive with other variants from the same subtype.

The study used vaccine-induced T cells are very interesting and valuable for HCV vaccine development, but the data of DC priming model are limited for evaluation of epitopes since the T cells were induced in the samples from one or two individuals.

Response: We would like to thank to thank reviewer-1 for the generally positive comments. In fact T cells could be primed in all individuals, (with the single exception of DC13), with at least one of the NS3-1406 variants tested, and many individuals could be primed with multiple variants (e.g. DC2, DC5, DC6, DC8). This is best seen in Fig 2. Thus an experimental model such as this may have a role when there is a need to interrogate one epitope in detail.

Major comments:

1. Fig1C: only one subject showed the cross-reactivity with one variant (not two). This is quite different from other epitopes. The titration data showed the different spots between these variants (may be significant, no statistics analysis), suggesting that this difference may be critical in T cell recognition of virus-infected cells.

Response: In our assays one individual showed cross reactivity to one variant (volunteer 001-032 to KLSSLGLNAV), and one individual showed cross reactivity to two variants (volunteer 003-328 to KLSGLGLNAV and KLSSLGLNAV), albeit at a reduced magnitude. We have now made this clearer in the manuscript.

European Journal of Immunology

We agree that the NS3-1406 epitope is quite different from the other two epitopes evaluated, showing more variants at a population level, and minimal cross-reactivity to some of the variants by vaccine induced T cells. For vaccine design this is an important issue as this is a very dominant epitope. It is for exactly this reason that we evaluated this epitope in the DC in-vitro priming assay-to see if we could identify an alternative variant epitope at this site that could be used for priming broadly reactive T cells. We also agree that the difference in the variants may be critical for T cell recognition of virus infected cells and have added a sentence to this effect in the manuscript.

We have not performed a formal statistical analysis on the cross-reactivity of the NS3-1406 epitope as this would not be meaningful given the small sample groups. However, we feel that that ex-vivo data from vaccinated volunteers showing limited cross reactivity is very consistent between individuals tested, and that this data is strongly supported by the in vitro titration experiment. The titration curves are particularly convincing showing reduced cross-reactivity to the variant at all peptide concentrations in both individuals (001-032 suppl fig 1 and 003-328 fig 1)

2. The evidence of cross-reactive T cell induction in the DC priming experiment is weak because they were induced in only 2-3 for 95A and 1-2 for others.

Response: T cell lines were induced in 6 volunteers for 95A, 4 of whom showed cross-reactive responses (DC4, DC5, DC6 and DC8). Cross reactive T cell lines were also induced in 4 volunteers primed with 95H (DC2, DC4, DC5 and DC6), in 3 volunteers primed with 95C, in 2 volunteers primed with 95B and in 1 volunteer primed with 95I. Overall cross-reactive responses to one or more variants were induced in all but 2 volunteers (DC14 and DC15). The magnitude of T cell responses was convincing in this experiment, and taken in context with the in vivo data, would suggest that these results were a fair representation of the ability of T cells to target variant peptides.

Whilst not all volunteers responded in an identical way (similar to the ex vivo vaccine induced T cells) the overall pattern of response suggested that genotype 1a variants would prime T cells that at best cross-reacted with other 1a variants, and likewise a 1b variant would prime T cells that cross reacted with other 1b variants (but not 1a). The authors do not intend to suggest that all viral variants would be covered by a given epitope variant if included in a vaccine, but rather, that an HCV vaccine would target either genotype 1a or 1b viruses at this epitope (depending on the variant included in the immunogen).

Minor comments:
1. The method in Fig2 is not clear. ICS assay?
Response: This is a tetramer FACS assay. This is now corrected and the assay type is now clearly given in the figure legend.

European Journal of Immunology

Reviewer: 2

Comments to the Author

This paper discusses an extremely important issue in viral vaccine field, the cross-reactivity. The authors investigated the cross reactive potential of T cells induced by an HCV vaccine based on adenoviral (Ad) vectors, focusing on three previously described epitopes. The spread of this kind of approach is necessary to improve the coverage of vaccines against viruses that present a high diversity of protein sequences and, consequently, of epitopes. The experiments were adequately conducted and the obtained results demonstrate that the vaccine seems to provide good coverage for common populations variants from the epitope region NS31073 CVNGVCWTV in the immunized individuals. The NS31436 ATDALMTGY and NS31406 KLSGLGINAV epitopes do not stimulated a desirable cross-reactive response, mainly in relation to the NS31406 epitope, even considering the demonstrated immunogenicity in the variant contained in the vector (95A). The authors raised questions about future strategies as the use of both variants for NS1436 Y/F or the synthesis of cross-reactive NS31406 variants through rational epitope design to improve coverage and the efficiency of immunogens and its possible consequences in loss of immunogenicity.

Response: We would like to thank reviewer 2 for the comments. We agree that a spread of this kind of approach is needed since a detailed evaluation of cross reactivity at discrete epitopes is imperative for rational vaccine design, especially in highly diverse pathogens such as HCV.

I just have two comments to share with you:

On page 5, line 2, the epitope ATDALMTGY is duplicated.

Response: This has been corrected.

I recommend the use of identity rather than homology to describe that two positions in a sequence are shared.

Response: We agree and have changed homology to identity throughout the manuscript.

Reviewer: 3

Comments to the Author

This is a follow-up study of the authors' 2012 Science Translation Medicine report on a phase 1 trial of the adenovirus-based HCV vaccines to induce broad and sustained T cell responses. HCV is genetically variable and the induction of functional cross-reactive T cells will be essential in any working vaccines. In

European Journal of Immunology

this study, through database search and several in vitro assays, the authors investigated how viral genotypic and epitope sequence differences might influence the effectiveness of the vaccine. The authors first used several clinical samples from the STM study to show cross-reactive T cells to three NS3 epitopes were induced by vaccination. They then developed a scheme to stimulate naïve T cells in normal blood donors in vitro with the NS3 1406 peptide and variants, and observed cross-reactive T cells were elicited in some cases (~30%). Based on the results, the authors conclude that immunization with the candidate AdV-based HCV vaccines can generate cross-reactive T cells at immunodominant epitopes. This reviewer is not certain if the results directly support the conclusion. Vaccination with the AdV-based vaccine is a complex process involving successful delivery, expression, processing and presentation of the viral antigens, while the in vitro assays subjected the T cells to non-physiological conditions (possible overdose of cytokines, peptides and DC to T cell ratio). Even so, the conditions used in the in vitro assays showed only a fraction (<50%) of the blood samples produced cross-reactive T cells.

The reviewer found the in vitro assays intriguing but the link of the results to the AdV-HCV vaccine unconvincing. In another word, it may be more useful if the authors focus on the in vitro assays and the particular NS3 epitopes, and explain why they works only in some samples; Or the authors refocus to define and analyze cross-reactive T cells and T cell epitopes in the vaccines from the 2012 STM study.

Response: We have rewritten the abstract and also added text within the manuscript to better explain the rational for linking the analysis using the STM volunteers with the in vitro DC experiments. The DC experiments leads directly from the observations that the NS31406 epitope variant within the vaccine immunogen is not universally cross reactive and purposely focuses on this epitope for this reason. If we re-focused the manuscript on the in vitro assays only, the rationale for these experiments would not be apparent, and the data would still be subject to the same criticism that this is an in vitro assay. Alternatively, if we focused only on an analysis of cross-reactive T cells and T cell epitopes in the vaccines from the 2012 STM study then we will not answer the question that we address in this manuscript; is there a NS31406 epitope variant that is potentially more cross reactive for use in a future T cell HCV vaccine? For this reason we would like to maintain the link between the STM vaccinated patients and the DC in vitro data.

We agree that our data both ex-vivo and also in the DC experiments shows limited cross-reactivity between NS31406 variants and we note also that cross reactivity is generally viral subtype specific. This is now clarified both in the abstract and in the final conclusion of the revised manuscript. The fact that out ex-vivo and DC data concur gives us confidence in the conclusions. We do acknowledge however, that the in vitro assay (like any in vitro model) is an imperfect one and discuss this in the penultimate paragraph.

A number of areas in this manuscript can be improved:

(1) What are the criteria for selection/inclusion of the particular clinical samples in this study? Have they been prescreened for high T cell responses?

Response: In the STM study all patients were assessed using 6 pools of peptides spanning the vaccine immunogen in IFN-γ ELISpot assays. Clinical samples were randomly selected to include subjects with a broad range of magnitude of T cell responses (total response ranged from 368-2435 SFC/ 106 PBMC and the response to the peptide pool containing the epitope NS31406 ranged from 250-1738 SFC/ 106 PBMC . This is now apparent from the data that we have now added to table 1 in response to comment (2) below. We have also added the criteria for selection to the methods section (p12).

(2) Supplementary Table 1 should be converted to a standard table, and ELISpot data (total IFN-SFC and NS3-specific IFN-SFC be included in the table. This is important for understanding the other data.

Response: This has been done and the ELISpot data has been added.

(3) The Los Alamos database used by the authors to analyze HCV epitope diversity is outdated. Recent virus sequences should be included in the analysis to ensure the epitopes used represent circulating viruses.

Response: We are confident that these epitope variants are still circulating based on sequence data that we have in Ad vaccinated HCV infected patients at Oxford and have added a sentence to the manuscript to this effect. However, the Los Alamos database still represents the largest international database of HCV sequences available and to generate the population data we required a dataset of thousands that was not geographically constrained.

(4) It seems unsatisfactory that the results and discussion are limited to the peptide sequences involved (or no involved) in the induction of cross-reactive T cells. So much is known about antigen presentation on MHC and TCR and it will be useful to interpret and discuss the data whether the peptides and cross-reactive variants follow the general rules in antigen presentation, or not and the explanation.

Response: We agree that this is a very interesting area and we are clear in the discussion that antigen presentation is dependent on both MHC and TCR. However, in this short report the discussion is constrained by the word limit.

Peer review correspondence

Second Editorial Decision – 5 September February 2014

Dear Prof. Barnes,

It is a pleasure to provisionally accept your manuscript entitled "CROSS-REACTIVITY OF HCV SPECIFIC VACCINE INDUCED T-CELLS AT IMMUNODOMINANT EPITOPES" for publication in the European Journal of Immunology.

For final acceptance, please follow the instructions below and return the requested items as soon as possible as we cannot process your manuscript further until all items listed below are dealt with.

Please note that EJI articles are now published online a few days after final acceptance (see Accepted Articles: http://onlinelibrary.wiley.com/journal/10.1002/(ISSN)1521-4141/accepted). The files used for the Accepted Articles are the final files and information supplied by you in Manuscript Central. You should therefore check that all the information (including author names) is correct as changes will NOT be permitted until the proofs stage.

We look forward to hearing from you and thank you for submitting your manuscript to the European Journal of Immunology.

Yours sincerely,
Laura Soto Vazquez

on behalf of
Prof. Annette Oxenius

Editorial Office
European Journal of Immunology
e-mail: ejied@wiley.com
www.eji-journal.eu
